# Simultaneous Combined Major Arterial and Lumbar Plexus Injury During Primary Extra Lateral Interbody Fusion: Case Report and Review of the Literature

**DOI:** 10.7759/cureus.13701

**Published:** 2021-03-04

**Authors:** Vasileios K Mousafeiris, Vasileios Tsekouras, Panagiotis Korovessis

**Affiliations:** 1 Orthopedics and Traumatology, General Hospital of Patras "Agios Andreas", Patras, GRC

**Keywords:** lumbar spine, xlif, llif, interbody fusion, major vascular injury, aorta, lumbar plexus, aorta injury, mesh stent

## Abstract

Extra lateral interbody fusion (XLIF) has been established in recent years as an effective approach to address degenerative lumbar disc disease (DLDD). Although neurological and vascular complications during XLIF have been reported, to our knowledge, a combination of simultaneous vascular and neurovascular complication during XLIF has not been reported to date.

A 72-year-old female patient was admitted to our orthopaedic department because of back pain associated with severe neuropathic radicular pain to her both lower extremities, incomplete paraplegia and low back fistula with serous secretion for several weeks. She had been wheel-chair bound since nine years before her admission in our department when she had her initial XLIF operation in another institution. Intraoperatively, an aorta lesion occurred, which was emergently addressed, along with lumbar plexus injury. Since then, she had an extensive history of subsequent operations that ended with a T10-S1 posterior lumbar fusion, with no improvement of her neurological condition, complicated by hardware-induced infection. She underwent her last operation in our department; removal of the posterior lumbar construct and extensive debridement of the posterior lumbar spine.

We present this rare case and we perform an extensive literature review. Although XLIF has been established in recent years, the report of major vascular injuries, although rare, has questioned its safety profile. Spine surgeons should be aware of catastrophic major neurovascular complications associated with this procedure and be prepared to address them.

## Introduction

Degenerative lumbar disc disease (DLDD) is a common condition affecting millions of people worldwide [1]. Minimally invasive (MI) surgical techniques including extreme lateral interbody fusion (XLIF), lower lumbar interbody fusion (LLIF) and direct lumbar interbody fusion (DLIF) have been proposed as safe and effective MI surgical approaches to treat DLDD, as they provide direct and good visualization of the lateral lumbar spine while reducing the rate of neurological, vessel and soft-tissue injuries. However, MI surgical approaches been accused of either lumbar plexus injuries, and in very rare cases, for life-threatening major vascular injuries [2-4].

To date, mostly neurological complications associated with lumbar plexus injury, usually temporary, have been reported [2-4]. Lumbar plexus injury has been reported as 13.28% as an average in various studies [2]. However, vascular complications involving both major and minor vessels have been reported on occasion during MIS lateral interbody fusion approaches [2, 4]. There are very few papers that describe minor vascular complications; however, the research works that report major vascular injury are even less. A recent systematic review reported 0 to 0.4% incidence of major vascular injury during XLIF or MI XLIF [4]. 

Although isolated neurologic or vascular complications have been reported in the literature, to our knowledge, simultaneous major vascular injury and lumbar plexus injury have not been presented in association with primary XLIF. We hereby present the first case of simultaneous abdominal aorta laceration and lumbar plexus injury during a left-sided primary XLIF surgery. A narrative review of the related literature is also discussed.

## Case presentation

A 72-year-old female patient was admitted to our outpatient department complaining of back pain associated with severe neuropathic radicular pain to her both lower extremities, incomplete paraplegia at the levels of L5 and S1 and low back fistula with serous secretion since several weeks. The patient had extensive surgical and medical history in another hospital and brought us all the medical reports from her previous admissions.

**Figure 1:**
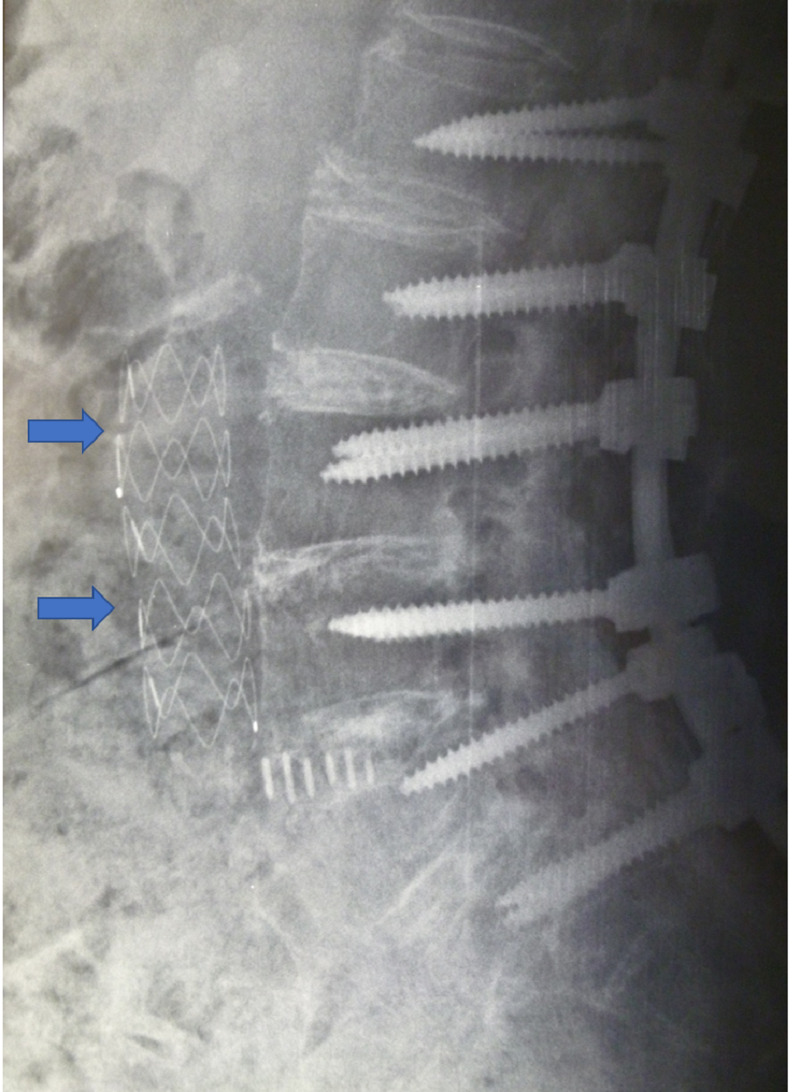
Lateral X-ray of the lumbar spine (sitting) on admission showing the instrumented lumbar spine. Intraluminal endovascular abdominal aorta mesh stent in situ (arrows).

Nine years before her admission to our department, she had undergone several anterior and posterior lumbar spine surgeries for L3-L5 spinal stenosis and neurologic claudication. The first operation had occurred at the age of 63 years. It was an anterior decompression and interbody fusion L3-L4, via a left-sided XLIF approach with neuromonitoring. While placing the intervertebral cage at the segment L4/L5, there was a pool of blood coming from the surgical site and the patient soon became hemodynamically unstable. The vascular surgeon on-site applied a hemostatic agent immediately along with packing and the decision to abort the posterior planned stabilization was made. Emergent angiography was performed, as the suspicion for major vascular injury arose; it showed laceration of the terminal aorta along with large expanding hematoma pressing on the lower abdominal aorta and the right common iliac injury. Following angiography, an intravascular mesh stent was successfully inserted by the interventional radiologist on site (Figure 1). The patient became hemodynamically stable and repeat angiography showed no blood escape from the aorta laceration site. Immediately postoperatively, the patient complained of decreased motor strength in all muscles below the knee (L5, S1) bilaterally and severe neuropathic pain. The patient remained stable and the decision to proceed to the aborted XLIF was made. Two months after the initial L4/L5 XLIF, the surgeon proceeded to the posterior percutaneous MIS stabilization.

**Figure 2:**
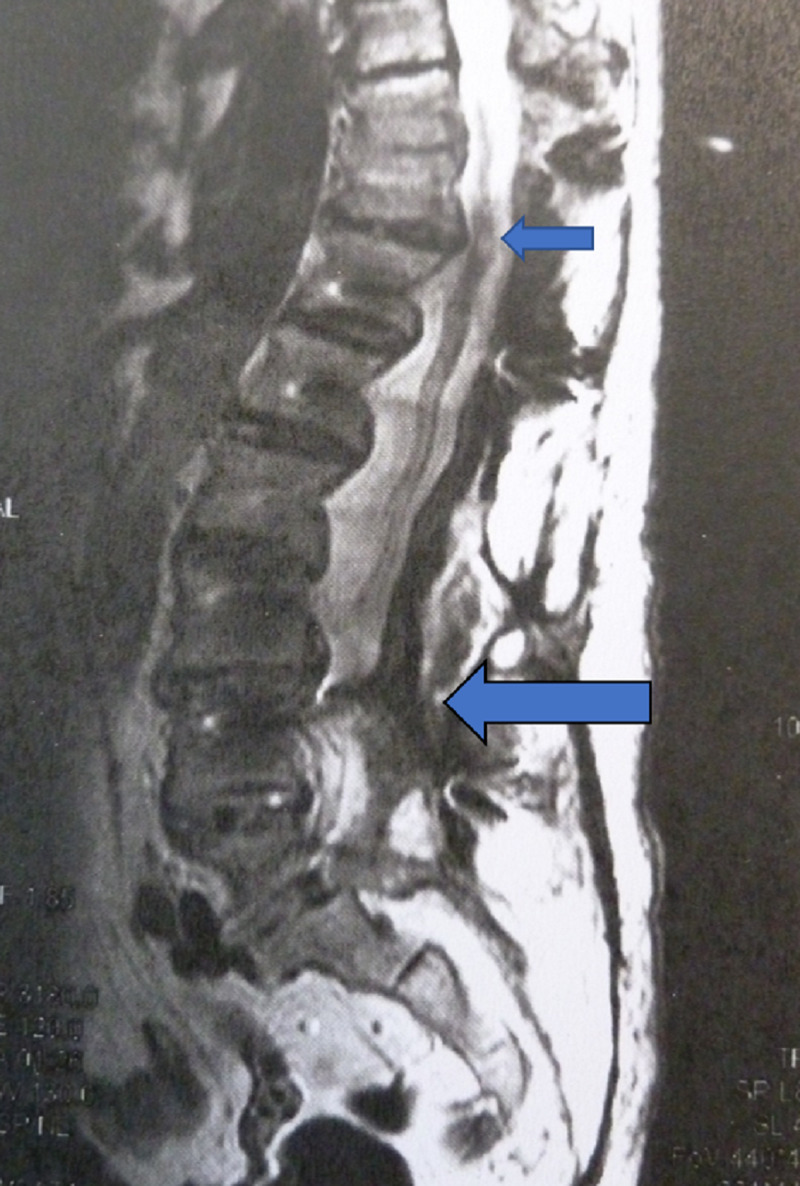
Lateral MRI showing myelopathy in the spinal cord at the level T11-T12 level (blue small arrow), Note significant dural adhesions at the level L3-L4 following MIS posterior decompression and fusion performed after primary XLIF (blue big arrow)

The subsequently performed CT and MRI of the thoracolumbar spine disclosed a significant spinal stenosis with myelopathy signs at the level T11-T12, so that the previous surgeons advised a wide decompression in the lower thoracic spine since it was considered a main source of the persistent lower extremities pain and neurologic deficit (Figure 2). 

**Figure 3:**
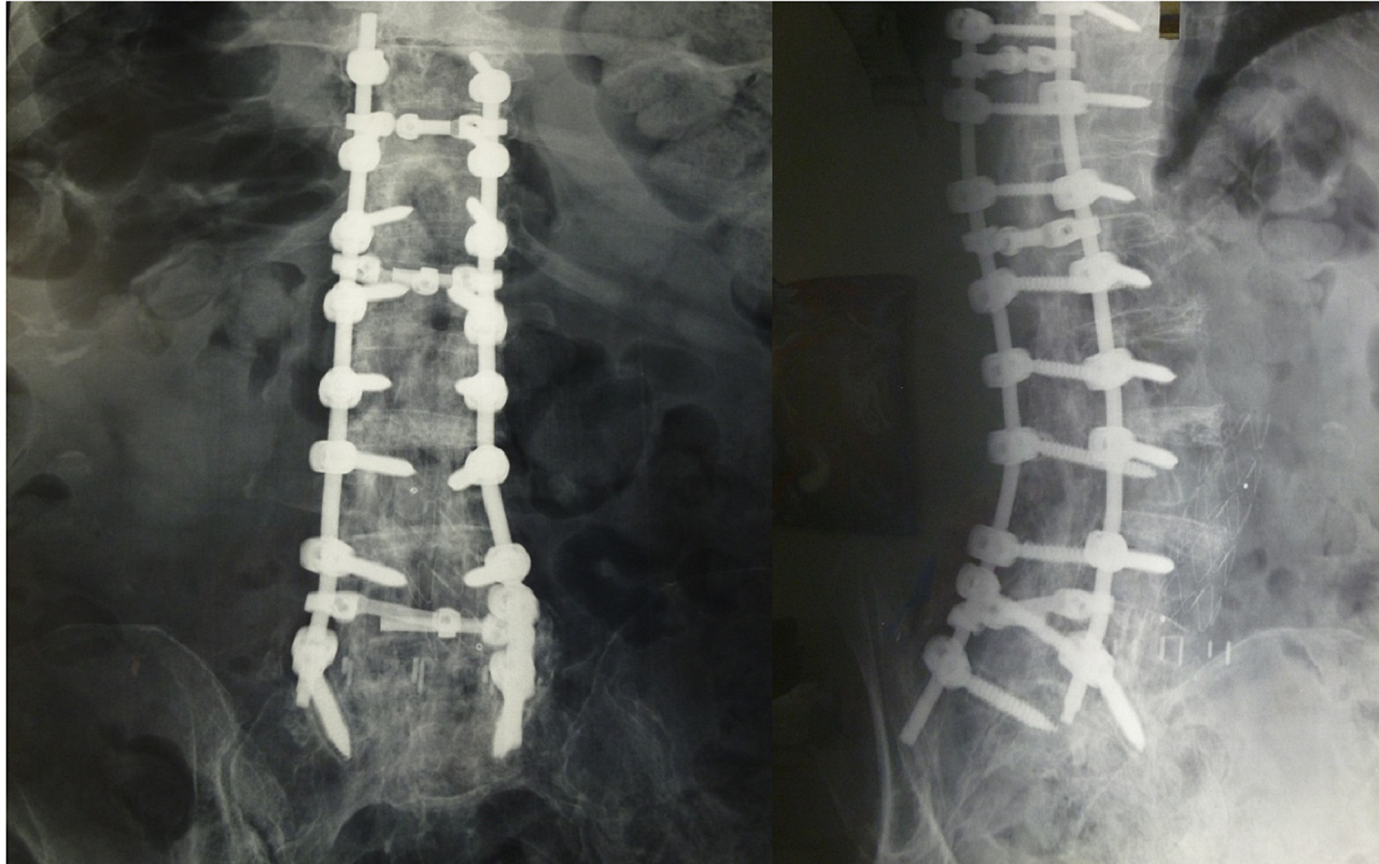
X-ray imaging (AP and Oblique views) of the lower thoracic and entire lumbar spine shows posterior spinal fusion at the level of T10-S1

**Figure 4:**
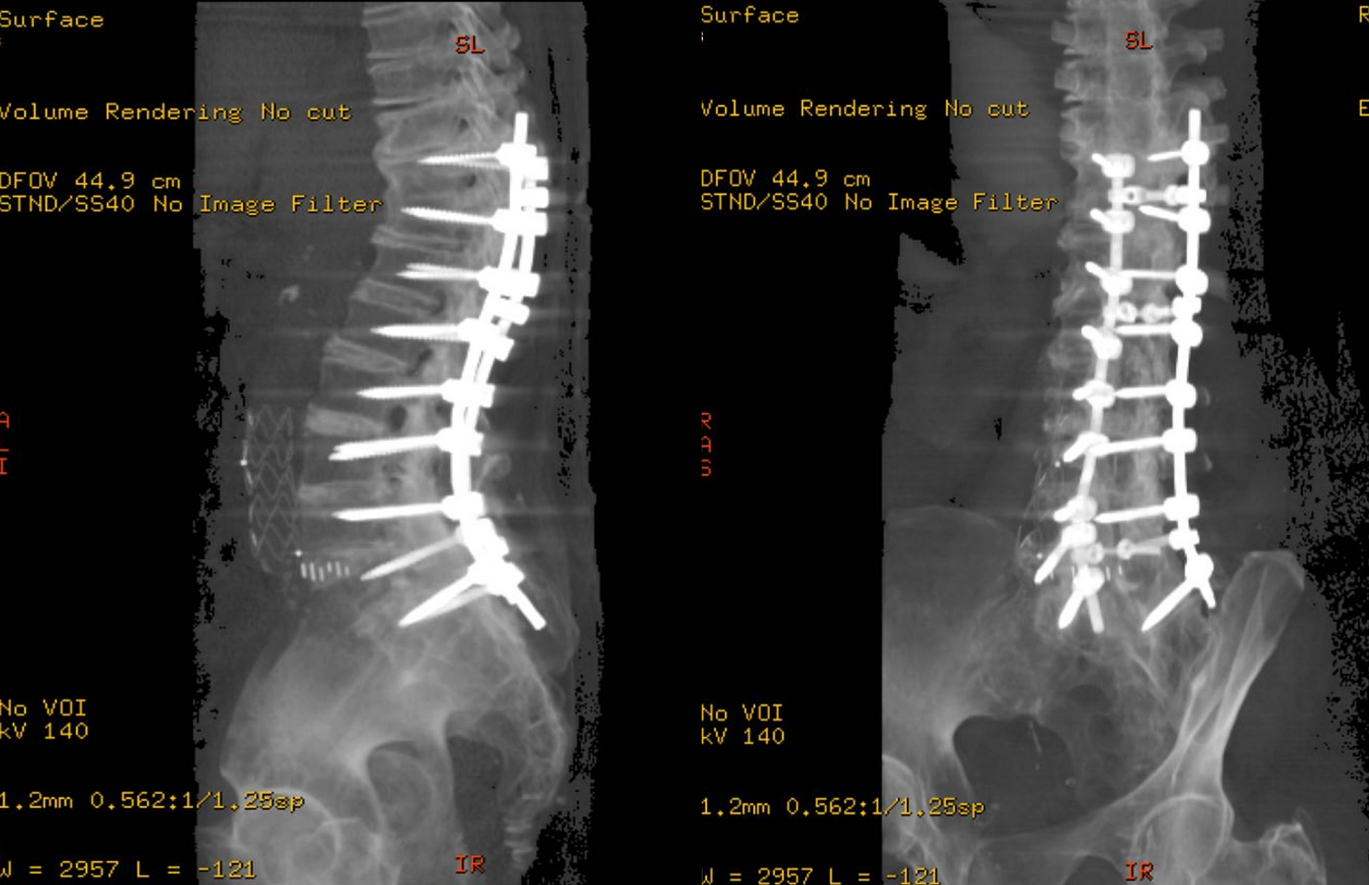
Computerized Tomography 3D reconstruction of the lower thoracic and lumbar spine showing completed fusion T10-S1 posterior lumbar fusion with simultaneous interbody in all fused segments

Therefore, a wide posterior decompression, including laminectomy and facetectomy was performed by the same surgeons at the level of T11-L3 with posterior pedicle screw fixation and fusion from T10-S1 levels (Figures 3-4).

On admission to our outpatient clinic, the patient was mobilized using a wheelchair and claimed severe pain in the lower extremities. She had been in pain-relief protocol with pethidine and morphine in a private pain clinic by an experienced anaesthetist with only temporary relief. Physical examination revealed a patient with marked muscular atrophy in both lower extremities and flexion contracture in her right knee of 30 degrees associated with severe osteoarthritis. There were two draining sinuses emerging from the back over the old posterior midline surgical scar. The lateral (XLIF) scar in the left side was without signs of infection. Neurovascular examination revealed motor deficit in the lower extremities as follows: iliopsoas bilaterally 3/5; quadriceps bilaterally 3/5; foot dorsal extensors and flexors bilaterally 1/5 and 2/5 respectively. Furthermore, sensation from L1 to S2 was decreased, worse at the levels of L4-S1 bilaterally. Therefore, only minor improvement was seen compared to the situation after the multiple operations in the first institution.

**Figure 5:**
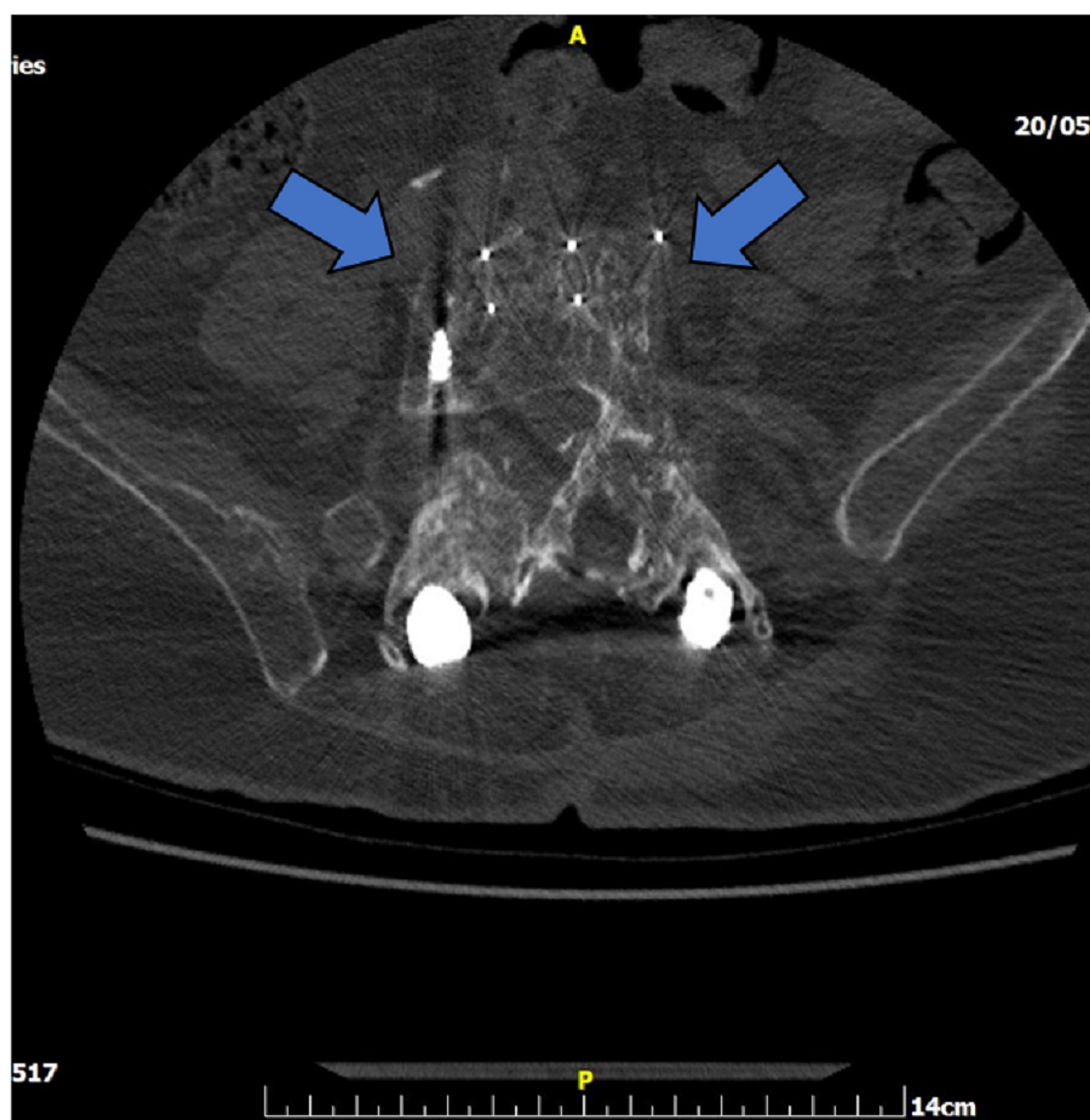
Computerized tomography (axial view) of the lower lumbar spine showing nice fusion within the cage and vertebral bodies L4-L5

**Figure 6:**
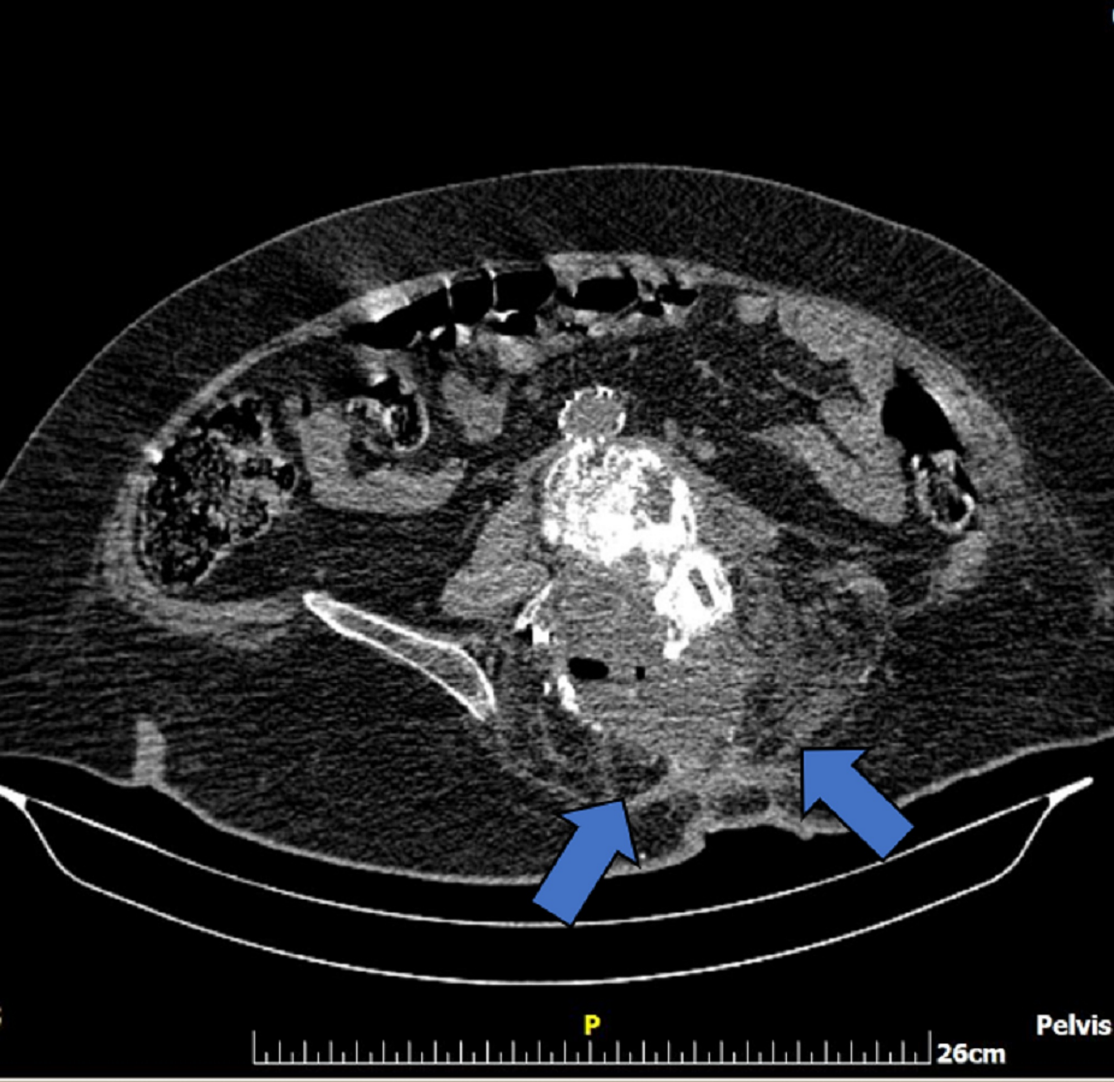
Axial view of Computerized Tomography in lower lumbar spine showing subfascial abscess formation extending to the posterior spinal elements (Arrow)

Wound cultures from the sinuses were collected on admission that disclosed E. coli and intravenous antibiotics were started. A CT showed completed fusion in all instrumented segments (Figures 4-5), but also revealed remarkable abscess formation underneath the lumbar fascia (Figure 6).

**Figure 7:**
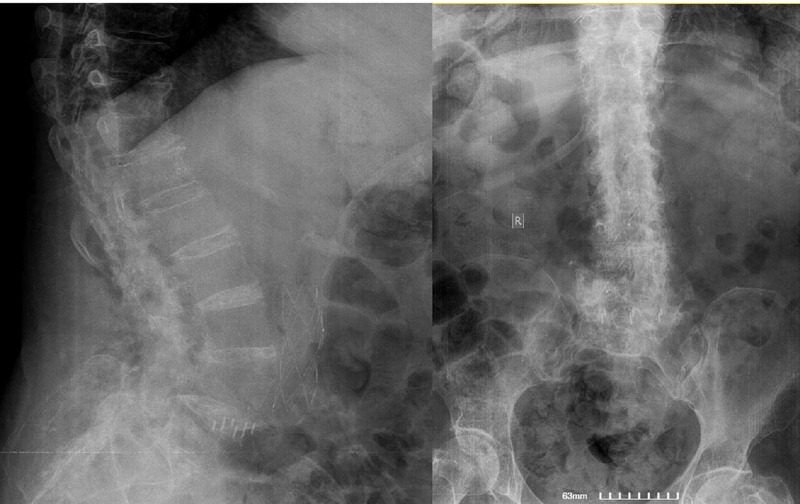
AP and lateral view of the lower thoracic and entire lumbar spine after the removal of the entire posterior fusion T10-S1 construct, which was the patient’s last operation

During her admission in our department, she underwent a posterior revision surgery from T10 to S1 including removal of the fistulae that emerged from underneath the deep lumbar fascia. Pus was draining from the subfascial area and was drained and debrided meticulously. No findings of meningocele or pseudomenigocele were disclosed elsewhere. Complete removal of the posterior spinal implants (screws, rods etc.) was performed since the fusion was completed and a chronic deep infection persisted (Figure 7).

Tissue samples taken from the posterior lumbar surgical site were cultured. Tissue culture grew E. coli and Pseudomonas strains. The antibiotic scheme was adjusted and continued for a total of four weeks until the patient was discharged; oral antibiotics followed for additional two months after discharge. She remained stable during her hospitalization, repeat blood cultures were negative and the patient was finally discharged to a rehabilitation facility.

In the first six months following this surgery, the patient reported slow but gradual improvement of her lower extremities neuropathic pain. Thirty months following this last surgery the patient was admitted again to our outpatient clinic. She was almost pain-free and was mobilizing with leg braces for the lower extremities. The ESR was 12 mm/1st hour and CRP was 0.5, within normal limits (<0.5). 

## Discussion

XLIF has been established as MI surgery that provides effectiveness in treating DLDD with less trauma, no bleeding and short hospital stay. More specifically it is proposed that XLIF would limit the major vascular and sympathetic plexus injuries associated with anterior lumbar interbody fusion (ALIF) as it has a lateral entry point [5-7]. However, as with every surgical procedure, XLIF, although rarely, may be associated with even serious complications [2-4]. Although most of the reported complications are neurologic, there are vascular complications reported as well [2-4]. However, to our knowledge, no studies with available follow-up exist, that report the combination of neurovascular complications following primary XLIF surgery.

We, hereby, present the first case with a combination of abdominal aorta laceration along with lumbar plexus injury during a left-sided L4-L5 primary XLIF procedure, with long-term follow up, in a 63-year-old woman. This is also only the second case of intraoperative abdominal aorta injury in the existing literature. We believe this case is of scientific importance for spine surgeons to show the potential complications following XLIF approach for spinal fusion.

In contrary to the surgeons’ anticipation with XLIF MIS approach for spinal stenosis, the patient had a complicated intraoperative and postoperative course with potential fatal great vascular and plexus injury and severe neuropathic pain in the lower extremities. We believe that both the major vascular injury and the neurological complication due to lumbar plexus injury occurred intraoperatively during the initial XLIF surgery. As a consequence, chronic deep infection followed that led to hospital admissions and ultimately to three additional surgeries.

We have been following the patient for three years after our revision surgery, and 12 years after the initial XLIF procedure that was performed in another institution. After the initial XLIF and the aorta laceration repair with the mesh stent the patient underwent a series of posterior spinal reoperations until our last operation, where we revised the spine for deep infection and fistula and removed the posterior hardware. No infection recurrence was seen until her last follow up almost three years after her last procedure.

XLIF has been established in recent years as a safe and effective procedure to treat degenerative spinal disorders. However, it is accused for increased neurological and vascular complications and also increased morbidity and mortality [2, 3]. Epstein et al., in a systematic review, reported 13.82% overall incidence of neural plexus injury, sensory deficits 0-75% (permanent in 62.5%) and motor deficits 0.7-33.6% [2, 3]. They also reported 0% to 0.03% to 0.4% incidence of major vascular injury during primary XLIF or MI XLIF [4]. Major vascular injury treatment options include open and percutaneous intraluminal stent placement, with the latter tending to become the gold standard for treating traumatic aortic injuries [8, 9].

Vascular injuries involving great arteries and veins during a primary XLIF procedure are rare, though have been described [10-13] (Table 1). However, there is no simultaneous neurovascular injury directly related to the XLIF operation reported in these cases. Santillan et al. in 2010 reported the first major vascular complication; an “iatrogenic” lumbar artery pseudoaneurysm after a L4-L5 XLIF [10]. The first major arterial complication by XLIF surgery was reported in 2015. They reported an intraoperative lower abdominal aorta rupture at the level of the terminal aorta that was repaired via an exploratory laparotomy [11]. The immediate postoperative course was uneventful and the patient was discharged home. After discharge, the patient had a fall and developed neurological symptomatology with back pain and inability to stand up because of L4-L5 cage dislodgement that, however, was not revised. In the two-year follow up, the patient showed marked improvement in her radicular symptoms. To our knowledge, this is the only case report with a possible combination of major vascular injury and neurological deficit, that is most likely, however, unrelated. Although the aorta injury occurred intraoperatively, the neurological complications developed most likely after the fall that the patient had after discharge. Furthermore, compared to our patient who had multiple operations and was wheelchair-bound, their patient had less severe neurological complications that did not require further operations and also improved in the course of time. The first fatal major vascular complication during a primary XLIF at the level of L4-L5, was reported in 2014. The authors reported an inferior vena cava rupture complicated by septic shock, multiorgan failure and subsequent death. Their patient had been reoperated multiple times due to iatrogenic complications that led to severe infection requiring readmission, like in our case. However, unlike in our case, their patient was reoperated only within four weeks after the initial XLIF surgery and also the infection progressed to septic shock, multiorgan failure and death. This case also does not report any lumbar plexus or neurological deficit [12]. In 2016, Buric et al. reported another case of direct injury and immediate repair of the common iliac vein during an L4-L5 primary XLIF [13]. 

**Table 1:** Case report studies with reported major vascular injuries during XLIF XLIF: Extra lateral interbody fusion; LLIF: lower lumbar interbody fusion

Author	Description	Type of study	Nr. of Patients (Nr. of levels)	Type of Surgery	Indications for surgery (Nr. of cases)	Nr. of vascular events	Treatment approach to address vascular injury	Other Data
Santillan et al. [10], 2010	= First major vascular injury	Case report	1	XLIF	Not available	“iatrogenic” lumbar artery pseudoaneurysm	Pseudoaneurysm was obstructed with two coils; patient hemodynamically stable, no transfusion needed, discharged home two days later	No lumbar plexus or neurological deficit reported, no follow up
Assina et al. [11], 2014	= First fatal major vascular injury	Case report	1	XLIF	Not available	Inferior vena cava rupture at L4-L5	Exploratory laparotomy with balloon occlusion of the IVC, massive transfusion protocol, five subsequent operations in the next four weeks; complicated by septic shock, multi-organ failure and death	The first reported fatal vascular XLIF complication / L4-L5 level, no lumbar plexus or neurological deficit reported, 29x pRBC transfusion, ICU admission, died of septic shock
Aichmair et al. [13], 2015	= First major arterial injury	Case report	1	LLIF	Lower back pain, adult scoliosis, multilevel disk space narrowing	Aorta injury (posteromedial aspect of the terminal aorta)	Exploratory laparotomy – postoperatively, the patient had a fall and resulted in L4-L5 cage anterior dislodgement that led to radicular symptoms (back pain and inability to stand up straight) that improved over two-year follow up;	L4-L5 level, follow-up period (two years): there was no further cage dislodgement, and the patient did not undergo any further lumbar spinal surgery
Buric et al. [12], 2016		Case report	1	XLIF	Spondylolisthesis L4-L5	Common iliac vein injury and repair	Bleeding site was clamped; bleeding stopped intraoperatively and XLIF was abandoned; patient transferred to ICU, discharged home eight days postoperatively	L4-L5 level, ICU, no lumbar plexus or neurological deficit reported, no follow up

Few case series and systematic reviews have investigated the number of vascular complications during primary XLIF or LLIF and even fewer authors have reported vascular complications [14-16] (Table 2). However, again, no combination of neurovascular complications was reported in the cases that suffered major vascular injuries. Kueper et al. [14] reported only one patient (0.056%) with intraoperative lower abdominal aorta rupture in a series of 900 XLIF patients. This case is most likely the same as reported from Aichmair et al., also in 2015 [13] (Table 2). Recently, a retrospective study [15] compared 1,772 patients and 2,709 patients that underwent the prepsoas and the transpsoas XLIF approach, respectively. The incidence of major vascular injury for the transpsoas group was statistically significantly lower than that of the prepsoas group (0.4% vs 1.8%, p=0.01). Manning et al. quite recently reported one patient (0.4%) with iliac vein injury in a series of 275 patients that underwent LLIF [16].

**Table 2:** Case series and systematic reviews reporting major vascular injuries during XLIF XLIF: Extra lateral interbody fusion; LLIF: lower lumbar interbody fusion; EXP: group where vascular/general surgeon was present

Author	Type of study	Nr. of Patients (Nr. of levels)	Type of Surgery	Indications for surgery	Vascular events	Treatment approach to address vascular injury	Other Data
Kueper et al. [14], 2015	Retrospective case series	900 (1,754)	LLIF	Not available	One case of aorta laceration at the L3/L4 level	Exploratory laparotomy – postop course uneventful	Aorta injury most probably the same case presented by Aichmair in 2015 4 other cases of minor vessel injuries (segmental arteries) / no lumbar plexus or neurological deficits of the vascular event case reported/ no follow up
Walker et al. [15], 2019	Systematic review and meta-analysis; Prepsoas: 20 studies vs. Transpsoas: 39 studies	6,481 (>10,000) – 1,772 prepsoas vs. 2,709 transpsoas approach (assessed for vascular injury)	XLIF	Inclusion criteria: studies had to have at least ten patients / Studies that did not mention complications or pseudarthrosis or subsidence were excluded	Major vascular injury: 21 events in prepsoas group (1.8%) vs. five events in transpsoas group (0.4%) – p=0.01	N/A	No reported cases of sympathetic plexus injury for transpsoas cases (95% CI 0.0–3.2) vs. reported rate of 5.4% (95% CI 2.2–12.6) in the prepsoas studies (p = 0.03); no relationship between vascular complication and neurological complication can be extracted
Manning et al. [16], 2020	Retrospective case series	275	LLIF	N/A	One case of iliac vein injury (0.4%)	Exploratory laparotomy – the vascular event occurred in the group that vascular/general surgeon was present (EXP group)	Presence vs. absence of vascular/general surgeon during the approach / no concomitant lumbar plexus or neurological deficits of the vascular event case

Although rare, vascular injuries during XLIF can be catastrophic, thus having a structured preoperative plan is essential. In line with that, the need for detailed anatomy assessment before the operation is crucial as retroperitoneal vessels show increased variability in their course, orientation and bifurcation height. Buric et al. proposed that all patients should have MRI imaging or even contrast MRI or angio-CT before an XLIF operation, in order to identify the position of the major vessels [12]. Berjano et al. proposed planning of docking points for XLIF to avoid major vascular injuries [17]. Walker et al. further delineated the need for preoperative vascular anatomy assessment [15]. Furthermore, Assina et al. suggest both MRI and CT imaging preoperatively, especially in the lower lumbar spine area as L4-L5 level is the most at risk for vascular injury [11].

The lower lumbar spine is also the area that commonly suffers from DLDD, so a vast number of XLIF surgeries occur between L3-L5. This area is also where the anatomy changes and makes it more prone to vascular and neural injuries. The space at the lower lumbar spine gets narrower; the great vessels move more posteriorly and laterally and as the neural elements move anteriorly [18]. Furthermore, in case of vascular complication during XLIF, the lateral incision is small enough that does not allow for adequate visualization of the vascular injury.

Although pre-operative planning is essential, it is also of critical importance to maintain a high index of suspicion for major vascular injuries intraoperatively, as they can lead to catastrophic or even fatal results [11]. Vascular complication during the XLIF should be suspected when there is a lot of blood lost in a short period of time or there are signs of hemorrhagic instability, such as tachycardia, hypotension etc. In case of serious vascular complication, the small lateral incision does not allow for adequate visualization to address the vascular injury. It is proposed that if 200-300 ml of blood are lost in a short period of time, then the surgeon should extend the incision in an attempt to identify the source of bleeding and should avoid blind attempts of hemostasis [12]. 

## Conclusions

XLIF or LLIF have been established as safe and effective procedures to treat spinal stenosis with DDD with experienced hands only. The majority of complications reported are non-vascular and mostly neurological. Although rare, vascular complications have been and can be catastrophic or even fatal. Furthermore, the combination of simultaneous neurovascular complications has not been reported to date. Therefore, it is important to assess the anatomy of the retroperitoneal space preoperatively through CT or MRI imaging in order to avoid vascular and neural injuries. Furthermore, it is critical to maintain high index of suspicion for injury to major vessels and to act accordingly. This case also reflects the notion that every procedure may come along with short-, mid- and long-term complications and to be prepared to address them.
